# Oxidative Stress in Human Toxicology

**DOI:** 10.3390/antiox10081159

**Published:** 2021-07-21

**Authors:** Tim Hofer

**Affiliations:** Department of Environmental Health, Norwegian Institute of Public Health, P.O. Box 222 Skøyen, N-0213 Oslo, Norway; tim.hofer@fhi.no; Tel.: +47-21076671

This Special Issue (same name as title) focuses on human exposure to foreign chemicals (xenobiotics) that cause oxidative stress. Certain xenobiotics and/or their metabolites (e.g., quinones) can directly mediate the formation of reactive oxygen species (ROS; e.g., superoxide and peroxides) [1] that can inflict damage to biomolecules [2,3] and/or affect signaling pathways. Xenobiotics can also indirectly cause oxidative stress by affecting protective proteins (i.e., antioxidant enzymes and metal transporters/chelators) and their expression (epigenetics). Increased ROS levels can cause antioxidant depletion. Some xenobiotics, however, are antioxidants [4,5]. Describing adverse outcome pathways (AOPs) related to toxicants and oxidative stress is presently a hot topic as well as suggesting relevant experimental models (e.g., what are suitable animals for studying human conditions, and, how can new 3D human cell culture systems be used) for detecting xenobiotics’ eventual toxic effects.

Drug-induced liver injury (DILI) is a serious challenge for pharmaceutical companies as DILI often appears post-approval in a minority of patients when the drug is administered to thousands of patients [6]. Oxidative damage can be a major mechanism in DILI. In their new review, M.T. Donato and L. Tolosa describe liver cell-based assays (e.g., using ROS-sensitive fluorescent probes) suitable for high-content screening to detect drug-induced oxidative stress in vitro [6]. The suitability of using traditional 2D liver cell monolayers [4] versus new 3D culture systems is also discussed.

Unborn (embryos and fetuses) and new-born babies often represent the most sensitive groups, but the reason for this is not always clear. T.B. Jeong et al. found that two-week-old weaning mice were more vulnerable towards carbon tetrachloride (CCl_4_)-induced liver injury than eight-week-old young adult mice [7]. Measured differences in basal hepatic glutathione (GSH (reduced form)) levels (higher in weaning mice) and the expression of hepatic enzymes such as cytochrome (CYP) P450s, GSH transferases, and GSH reductases may explain this age-related (developmental) susceptibility [7]. Adult mice had lower levels of the hepatic phase I enzymes CYP2E1 and CYP3A but had higher levels of phase II GSH S-transferase enzymes. CCl_4_ markedly lowered the hepatic GSH level only in weaning mice that suffered from various types of damages including oxidative stress-inflicted ones, e.g., from ROS and lipid peroxidation.

Vancomycin is an antibiotic used to treat bacterial infections. It is often administrated intravenously. A side effect is that it can cause kidney injury, i.e., Vancomycin-associated acute kidney injury (VAKI) for which oxidative stress is thought to be a main mechanism. In a clinical metabolomic study by H.–S. Lee et al. [8], serum concentrations of amino acids and amino acid derivatives/metabolites were compared between human patients who met VAKI criteria vs. non-VAKI subjects (divided into three subgroups, two of which did not receive Vancomycin). Significant differences among the groups were found for some measured parameters, particularly for the tryptophan (metabolized via the kynurenine pathway) metabolite serotonin (5-HT) and its metabolite 5-hydroxyindoleacetic acid (5-HIAA), as well as for the ratio 5-HIAA/5-HT. The authors conclude that their study demonstrates that an increased 5-HIAA/5-HT ratio has the potential to act as a novel biomarker for VAKI detection and that this ratio can be a useful indicator for oxidative stress and inflammation [8].

Silver nanoparticle (AgNP) interaction with cultured human adherent (lung epithelial A549) and suspension (monocytic U937) cells under static and dynamic flow conditions was investigated by K.E. Burns et al. [9]. The measured parameters included AgNP cell association, oxidative stress-related (intracellular ROS and GSH/GSSG ratio (GSSG is oxidized glutathione)) levels, activation of oxidative stress sensitive signaling pathways (phosphorylation of tumor suppressor protein p53 and nuclear factor kappa B (NFκB)), and production of inflammatory cytokines (interleukin-1β (IL-1β) and tumor necrosis factor-α (TNF-α)). AgNP cell association was higher for A549 than for U937 cells. For adherent cells, dynamic flow reduced AgNP contact due to the disruption of sedimentation. For suspension cells, however, dynamic flow increased AgNP cell association. Oxidative stress levels were found to be a function of cell type and flow conditions. U937 monocytes secreted considerably more cytokines (also when untreated) than alveolar epithelial A549 cells [9].

Arabic gum (*Acacia senegal*) is a plant extract known to have antioxidant and cytoprotective properties [10]. Using young adult rats, I. El–Garawani et al. investigated if Arabic gum, dissolved and administrated daily through drinking water, protected against oxidative stress and damage from a single intravenous high dose of Ioxitalamate (Telebrix-35^®^), an iodinated contrast medium used for X-ray imaging. Ioxitalamate caused various toxicities. Apoptosis in kidneys and blood leucocyte cytotoxicity were more severe after 14 days than after one day of Ioxitalamate injection. Arabic gum restored the oxidative stress status in kidneys, exerted antigenotoxic potential against DNA damage in peripheral blood leucocytes and bone marrow, and protected against bone marrow mitotic index alterations, results that can be attributed to metabolites of Arabic gum [10].

The emission of mercury (Hg) into the atmosphere, e.g., during power plant coal burning, leads to widespread global Hg contamination. After settlement, microbes convert Hg into methylmercury (MeHg) that possesses an increased lipophilicity (it readily crosses the placenta and concentrates in the fetal brain [11]) and neurotoxicity with distinct fetal and adult clinical forms. In their new review, M. Fujimura and F. Usuki describe several mechanisms of how MeHg mediates cellular toxicity [12]. Not only does MeHg disturb antioxidant proteins and enzymes, causing oxidative stress due to its high affinity for selenohydryl (SeH) and sulfhydryl (SH) groups as well as selenides (Se-Se), but MeHg also prevents the synthesis (Se-dependent) of selenoenzymes due to mRNA degradation, affects cellular signaling pathways such as those involving redox-regulated Kelch-like ECH-associated protein 1 (Keap1), and, induces neuronal hyperactivity and apoptosis [12].

A majority (85–90%) of Alzheimer’s and Parkinson’s disease cases are thought to result from nongenetic causes. Thus, environmental factors including chemical exposures may play strong roles. Post-mortem analyses of affected brain regions often reveal oxidative damage. These diseases develop with age and bioaccumulation of foreign chemicals (xenobiotics) [13] is suspected. A dark brown pigment present in catecholamine neurons called neuromelanin binds xenobiotics (both organic substances and metals) as well as endogenously formed potentially toxic metabolites (e.g., dopamine oxidation products) and excess metals (e.g., iron) that are stored away inside membrane-encapsulated tiny balls. The neuromelanin content increases almost linearly with age inside dopaminergic and noradrenergic brain neurons. Old neurons are sensitive to toxic insults and are lost in disease. In their new review, A. Cappucciati et al. [14] hypothesize that xenobiotics somehow disturb the build-up of neuromelanin and/or its protective function, which causes toxicity (including ROS formation) as xenobiotics and endogenous metabolites and excess metals are no longer stored away. The authors review the latest knowledge on neuromelanin biosynthesis, list neuromelanin-interacting xenobiotics, and suggest suitable experimental models for studying neuromelanin-related toxicity [14]. The authors question the use of rodents such as mice and rats (devoid or low in neuromelanin) as models when resembling human (high in neuromelanin) degenerative diseases.

In conclusion, oxidative stress from xenobiotic exposure is relevant for various human conditions and their induction. Identifying, developing, and using increasingly more suitable experimental models may be key to success in human toxicology research.
